# Iron Rims in Patients With Multiple Sclerosis as Neurodegenerative Marker? A 7-Tesla Magnetic Resonance Study

**DOI:** 10.3389/fneur.2021.632749

**Published:** 2021-12-21

**Authors:** A. Dal-Bianco, R. Schranzer, G. Grabner, M. Lanzinger, S. Kolbrink, G. Pusswald, P. Altmann, M. Ponleitner, M. Weber, B. Kornek, K. Zebenholzer, C. Schmied, T. Berger, H. Lassmann, S. Trattnig, S. Hametner, F. Leutmezer, P. Rommer

**Affiliations:** ^1^Department of Neurology, Vienna, Austria; ^2^Department of Medical Engineering, Carinthia University of Applied Sciences, Klagenfurt, Austria; ^3^Department of Biomedical Imaging and Image-Guided Therapy, High Field Magnetic Resonance Centre, Vienna, Austria; ^4^Department of Neuroimmunology, Center for Brain Research, Vienna, Austria; ^5^Institute of Neurology, Medical University of Vienna, Vienna, Austria

**Keywords:** multiple sclerosis, rim lesions, iron, atrophy, serum neurofilament light chain, neuropsychological impairment, 7 Tesla (7T), MRI

## Abstract

**Introduction:** Multiple sclerosis (MS) is a demyelinating and neurodegenerative disease of the central nervous system, characterized by inflammatory-driven demyelination. Symptoms in MS manifest as both physical and neuropsychological deficits. With time, inflammation is accompanied by neurodegeneration, indicated by brain volume loss on an MRI. Here, we combined clinical, imaging, and serum biomarkers in patients with iron rim lesions (IRLs), which lead to severe tissue destruction and thus contribute to the accumulation of clinical disability.

**Objectives:** Subcortical atrophy and ventricular enlargement using an automatic segmentation pipeline for 7 Tesla (T) MRI, serum neurofilament light chain (sNfL) levels, and neuropsychological performance in patients with MS with IRLs and non-IRLs were assessed.

**Methods:** In total 29 patients with MS [15 women, 24 relapsing-remitting multiple sclerosis (RRMS), and five secondary-progressive multiple sclerosis (SPMS)] aged 38 (22–69) years with an Expanded Disability Status Score of 2 (0–8) and a disease duration of 11 (5–40) years underwent neurological and neuropsychological examinations. Volumes of lesions, subcortical structures, and lateral ventricles on 7-T MRI (SWI, FLAIR, and MP2RAGE, 3D Segmentation Software) and sNfL concentrations using the Simoa SR-X Analyzer in IRL and non-IRL patients were assessed.

**Results:** (1) Iron rim lesions patients had a higher FLAIR lesion count (*p* = 0.047). Patients with higher MP2Rage lesion volume exhibited more IRLs (*p* <0.014) and showed poorer performance in the information processing speed tested within 1 year using the Symbol Digit Modalities Test (SDMT) (*p* <0.047). (2) Within 3 years, patients showed atrophy of the thalamus (*p* = 0.021) and putamen (*p* = 0.043) and enlargement of the lateral ventricles (*p* = 0.012). At baseline and after 3 years, thalamic volumes were lower in IRLs than in non-IRL patients (*p* = 0.045). (3) At baseline, IRL patients had higher sNfL concentrations (*p* = 0.028). Higher sNfL concentrations were associated with poorer SDMT (*p* = 0.004), regardless of IRL presence. (4) IRL and non-IRL patients showed no significant difference in the neuropsychological performance within 1 year.

**Conclusions:** Compared with non-IRL patients, IRL patients had higher FLAIR lesion counts, smaller thalamic volumes, and higher sNfL concentrations. Our pilot study combines IRL and sNfL, two biomarkers considered indicative for neurodegenerative processes. Our preliminary data underscore the reported destructive nature of IRLs.

## Introduction

**Figure 1:**
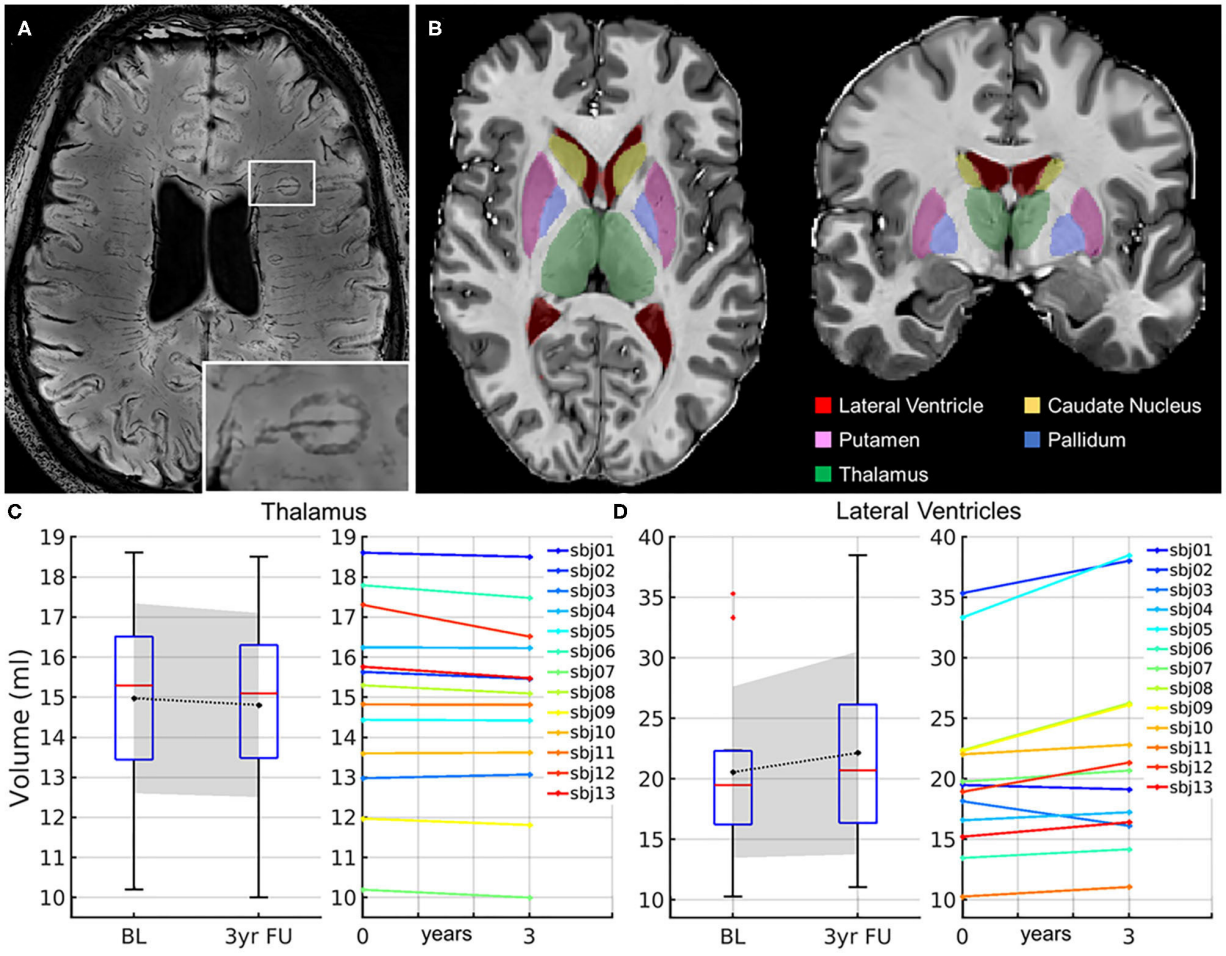
Images scanned at 7T. **(A)** FLAIR-SWI overview image shows multiple hyperintense white matter lesions surrounded by SWI-detected hypointense iron rims in a 27-year-old male relapsing-remitting multiple sclerosis (RRMS) patient with 7 years of disease duration and a Multiple Sclerosis Severity Score (MSSS) of 6.98. The white rectangle indicates the magnified iron rim lesion (IRL). **(B)** Segmentation of the subcortical deep gray matter structures. Subcortical segmentation maps are overlaid on the 7T T1-weighted MP2RAGE single-subject template from a male RRMS patient. **(C)** Significant reduction of the thalamus volume within 3 years within the cohort (left) and according to individual courses (right). **(D)** Significant enlargement of the lateral ventricles of the cohort (left) and according to individual courses (right). BL, baseline; FU, follow-up; ml, milliliter; sbj, subject; yr, year.

Multiple sclerosis (MS) is a chronic disease of the central nervous system with focal and diffuse inflammation within the white and gray matter (1, 2) leading to demyelination and whole-brain atrophy (3) due to destruction and loss of myelin, oligodendrocytes, and axons (4, 5). Worldwide, an estimated 2.5 million people suffer from MS. Most people with MS are diagnosed between the age of 20 and 40. Chronic tissue destruction over years leads to disability and cognitive impairment of varying degrees in patients. Serum and imaging biomarkers are becoming more important for the prediction of disease severity at an already early stage (6–15). It is, therefore, crucial to evaluate the clinical value of imaging and body fluid biomarkers. One such biomarker is the identification of iron rim lesions (IRLs) as shown in Figure 1A. About 50% of chronic active lesions are IRLs characterized by a susceptibility-weighted image (SWI) or R2^*^-detected hypointense or quantitative susceptibility mapping (QSM)-detected hyperintense rim of iron-containing microglia and macrophages representing their demyelination border (7, 15–17). They are associated with slow expansion (7, 9, 15) and less remyelination (17). IRLs are found in all clinical stages of MS (9, 18–23). They are particularly common in the transition phase from relapsing-remitting MS (RRMS) into secondary-progressive MS (SPMS) and are therefore considered as potential imaging markers of disease progression. They are found in around 50–66% of patients with MS (8, 24) and are also detectable at 3T (8, 25–29). IRLs show more pronounced T1-hypointensity and less remyelination compared to non-IRLs, suggesting the proinflammatory rim to mark for remyelination failure and/or irreversible tissue damage (15, 17) and show more myelin damage compared with non-IRLs (30–32).

The destructive nature of IRLs adds to the already agreed contribution of lesion burden in the white matter and the increasing cortical demyelination in the progressive stage (33) and is amplified by brain aging and mitochondrial damage (34). According to a recent clinical study, IRL patients showed a significantly higher lesion load and ventricular volume, whereas white matter and basal ganglia volumes were lower compared to non-IRL patients (8). Male patients showed a significantly higher than 10-fold risk for at least 1 IRL (35), and patients with more than 4 IRLs were described to reach the motor and cognitive disability significantly earlier and show a higher prevalence of clinically progressive MS compared to patients without IRLs (8). These clinical and imaging observations underscore the relevance of IRLs as an important imaging biomarker for progression with a more severe disease course.

The role of another biomarker, serum neurofilament light chain (sNfL), has attracted considerable interest as a parameter for axonal damage to monitor and predict disease progression (36–41). Elevated sNfL levels correlated highly with imaging disease activity (gadolinium positive signal on T1, GD^+^T1, and T2 lesion volume) and tissue destruction (brain atrophy) (36, 38, 42), and patients with RRMS and SPMS showed significantly higher levels of sNfL compared to healthy controls (13) and returned to normal levels after autologous hematopoietic stem cell transplantation (IAHSCT) (43).

A recent study pointed out a significant association between IRL and increased sNfL levels, independent of previously shown factors influencing sNfL, such as T2 lesion load, disease course, Expanded Disability Status Scale (EDSS), and disease-modifying therapy (DMT) (44). In addition, it has been reported that patients with MS with elevated sNfL levels have an increased risk of physical deterioration (13) and a neuropsychological decline in terms of processing speed (11), memory, and executive function (45). sNfL is reported to have a prognostic value for the conversion from clinically isolated syndrome to clinical-definite MS (46). Furthermore, sNfL levels assessed early from disease onset are reported to correlate significantly with 10-year follow-up MRI parameters, such as T2 lesion load and whole-brain atrophy and fatigue (14, 37, 47) and with long-term clinical motor outcome after 15 years (12) but only weakly with the long-term evolution of cognitive performance (48). Nevertheless, sNfL research is currently in its early stages, and a normative database of sNfL in healthy individuals is still needed including the effects of age and comorbidities (41, 49).

In this study, we report data from our long-term 7T follow-up cohort of 29 MS patients with 7T MRI-detected IRLs in terms of subcortical atrophy detected by 7T MRI, a long-term observation of sNfL, and neuropsychological performance.

## Patients and Methods

### Patients

**Figure 2:**
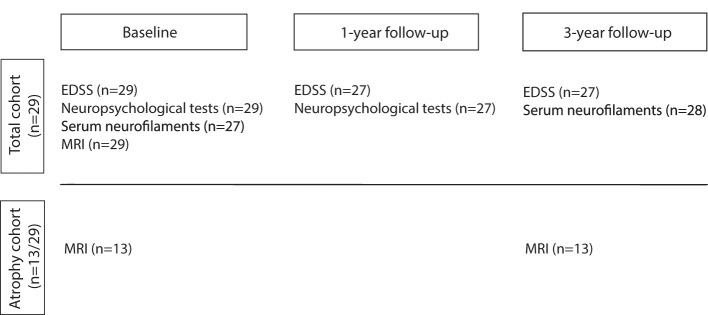
The flowchart provides a temporal overview of the examinations of the study cohort. Total cohort (*n* = 29) underwent examinations including Expanded Disability Status Scale (EDSS), Timed 25-Foot Walk Test (T25FW), Nine-Hole-Peg-Test (9-HPT), Symbol Digit Modality Test (SDMT), a blood draw for serum neurofilament light chain (sNfL), and 7T MR imaging (SWI, FLAIR, and MP2RAGE) according to the time point indicated above. Lateral ventricles and subcortical structures were measured only in the so-called atrophy cohort of 13 patients as they were scanned at both time points within 1 month, which increases data homogeneity. Two RRMS patients dropped out before 1-year follow-up examination due to time constraints and a move abroad.

Demographic, clinical, and MRI data of 29 patients from our long-term 7T MS follow-up cohort observed since 2010 (7, 15, 50) are shown in Tables 1, 2 according to the time points of this substudy. Patients were diagnosed with clinically definite MS according to the revised McDonald criteria (51) at the Department of Neurology, Medical University of Vienna, Austria. All patients met the following criteria at the time of inclusion since 2010: age >18 years, ERSS ≤ 6.5 (52), no corticosteroid therapy during the last 3 months preceding each MRI and blood sampling and no contraindications for 7T MRI. The study was approved by the local university ethics committee (EC 154/2009). Written informed consent was obtained from each patient. In total 29 patients (15 women, 24 RRMS, five SPMS) had a median age of 38 (range 22–69) years and a disease duration of 11 (range 5–40) years, an EDSS at baseline of 2.0 (range 0–8.0), and a Multiple Sclerosis Severity Scale (MSSS) at 3-year follow-up (3yr FU) of 1.8 (range 0.1–7.0) reflecting disease progression according to Roxburgh et al. (53). Since study inclusion in 2010, 2 SPMS patients exceeded the EDSS of 6.5. All patients underwent annual neurological examinations including medical history, EDSS, Timed 25-Foot Walk Test (T25FW), and Nine-Hole Peg Test (9-HPT). At study baseline, 48.3% of patients (14/29) received first-line DMT, 34.5% (10/29) received second-line DMT, and 17.2% (5/29) received no DMT. At the end of the observation period, therapy was discontinued in 2 SPMS patients [6.9% (2/29)] without superimposed relapses. Patients were neuropsychologically tested on the Symbol Digit Modalities Test (SDMT), a measure of cognitive information processing speed, which is a characteristic deficit in MS patients (*n* = 29) and 1 year thereafter (*n* = 27) (54). Annual 7T-MRI scans (SWI, FLAIR, and MP2RAGE) were used to assess lesion burden and 3yr FU volumetry of lateral ventricles and subcortical structures. Assessment of sNfL concentration was based on blood samples taken at baseline (*n* = 27) and after 3 years (*n* = 28). Two patients with RRMS withdrew from the study after baseline because of time constraints and relocation abroad, respectively. All time points of examinations are shown in Figure 2.

**Table 1:** Demographic, clinical, and radiographic data of the total study cohort.

	**Total cohort**	**IRL patients**	**Non-IRL patients**	***p*-values IRL vs. Non-IRL**
**Number of analyzed patients/with IRLs**	29	21	8	
**Gender (f/m)**	15/14	10/11	5/3	
**Number of IRL (f/m)**	–	166 (46/120)	–	
**Age, years**	38 (22–69)	38 (22–60)	36 (22–69)	
**Disease duration, years**	11 (5–40)	11 (5–37)	12.5 (6–40)	
**Disease course (RRMS/SPMS)**	24/5	17/4	7/1	
**EDSS**
Baseline^a^ (*n =* 29/21/8)^b^	2 (0–8)	2 (0–7.5)	1.5 (0–8)	
1yr FU^a^ (*n =* 27/19/8)^b^	2 (0–8)	2 (0–7.5)	2 (0–8)	
At sNfL measure 3yr FU (*n =* 27/19/8) ^b^	2 (0–8)	2 (0–8)	2 (0–8)	
**MSSS**
At 1yr FU^a^ (*n =* 26/19/7)^b^	1.3 (0.11–7)	1.4 (0.2–7)	1.1 (0.1–5)	
At sNfL measure 3yr FU (*n =* 26/19/7)^b^	1.8 (0.1–7)	1.7 (0.2–7)	2.8 (0.1–5)	
**Relapse count**
Baseline^a^ (*n =* 29/21/8)^b^	0 (0–2)	0 (0–2)	0 (0–1)	
1yr FU^a^ (*n =* 28/20/8)^b^	0 (0–2)	0 (0–2)	0 (0–1)	
**Treatment (none/first-line/second line)**
Baseline^a^	5/14/10	3/10/8	2/4/2	
1yr FU^a^	7/13/9	5/9/7	2/4/2	
**9-HPT, (r/l)**
Baseline^a^ (*n =* 28/20/8)^b^	20.9 (15.6–41.1)/22.8 (15.95–43.6)	21.4 (15.7–41.1)/23.3 (15.9–43.6)	20.08 (17.1–34.7)/21.2 (16.5–34.4)	
1yr FU^a^ (*n =* 27/19/8)^b^	20.4 (14.2–39.4)/21.9 (15.4–85.8)	20.2 (15.7–39.4)/21.9 (15.4–85.8)	21.1 (14.2–33.1)/19.8 (16.7–35.3)	
**T25FW**
Baseline^a^ (*n =* 27/20/7)^b^	5.5 (4.2–16.4)	5.4 (4.2–16.4)	5.6 (5.3–7.4)	
1yr FU^a^ (*n =* 25/18/7)^b^	5.9 (4.1–18.0)	5.8 (4.1–18.0)	6.0 (4.9–9.5)	
**SDMT** ***z*****-score**
Baseline^a^ (*n =* 29/21/8)^b^	0 (−3 to 1.5)	−0.5 (−3 to 1.5)	0 (−2 to 1)	
1yr FU^a^ (*n* = 27/19/8)^b^	0 (−3 to 2.5)	−0.5 (−3 to 2.5)	1.25 (−2.5 to 2)	
**sNfL (pg/ml)**
Baseline (*n =* 27/19/8)^b^	5.7 (3.2–23.7)	**7.6 (3.3–19.2)**	**4.4 (3.2–23.72)**	0.045
3yr FU (*n =* 28/20/8)^b^	8.0 (3.8–21.5)	8.8 (5.2–21.5)	6.1 (3.8–19.8)	
**FLAIR lesion count;** *n* = (29/21/8)^b^	30 (2–98)	**32 (5–98)**	**11.5 (2–64)**	0.047
**MP2RAGE non-IRL lesion count;** *n* = (29/21/8)^b^	20 (1–135)	23 (3–115)	11.5 (1–135)	
**MP2RAGE IRL lesion count;** *n* = (29/21/8)^b^	–	3 (1–41)	–	
**MP2RAGE Non-IRL lesion volume, ml;** *n* = (29/21/8)^b^	29.4 (0.8–439.9)	31.4 (2.4–241.9)	14.4 (0.8–439.9)	

*All values are given in median (range)*.

a *Values refer to the time point of the neuropsychological testing at baseline and 1yr FU*.

b *Numbers in brackets indicate the number of patients grouped according to the column categories (total cohort, IRL, and non-IRL)*.

*The p-values < 0.05 indicate significant results and are marked in boldface*.

*BL, baseline; EDSS, Expanded Disability Status Scale; f, female; FLAIR, fluid-attenuated inversion recovery; FU, follow-up; IRL, iron rim lesion; m, male; MP2RAGE, magnetization Prepared RApid Gradient Echo; MSSS, Multiple Sclerosis Severity Score; r, range; RRMS, relapsing-remitting multiple sclerosis; SDMT, Symbol Digit Modality Test; sNfL, serum neurofilament light chain; SPMS, secondary-progressive multiple sclerosis; SWI, susceptibility-weighted images; yr, year; 9HPT, Nine-Hole-Peg-Test; T25FW, timed 25-foot walk test; r/l, right hand/left hand*.

**Table 2:** Demographic, clinical, and MRI data of the atrophy cohort.

	**Total atrophy cohort**	***p*-values total atrophy cohort BL vs. 3yr FU**	**IRL patients atrophy cohort**	**Non-IRL patients atrophy cohort**	***p*–values IRL vs. Non-IRL**	***p*–values non-IRL BL vs. 3yr FU**
**Number of analyzed patients**	13		8	5		
**Gender (f/m)**	7/6		5/3	2/3		
**Number of IRL (f/m)**	58		37/21	**–**		
**Age, years; median (range)**	45 (22–53)		49.5 (25–53)	30 (22–49)		
**Disease duration, years; median (range)**	11 (5–19)		12.5 (5–19)	11 (6–16)		
**Disease course (RRMS/SPMS)**	13/0		8/0	5/0		
**EDSS; median (range)**						
Baseline^a^	1.5 (0–2.5)		1.5 (0–2.5)	1.5 (0–2.5)		
3yr FU^a^	2 (0.2.5)		1.75 (0–2.5)	2 (0–2.5)		
**MSSS; median (range)**
3yr FU^a^	1.4 (0.1–3.5)		1.4 (0.2–2.1)	2.8 (0.1–3.5)		
**Lateral ventricles, ml; mean (STD)**
Baseline^a^	**20.6 (7.1)**	0.012	22.6 (8.2)	**17.3 (3.5)**		0.045
3yr FU^a^	**22.2 (8.4)**		24.1 (9.8)	**19.1 (4.8)**		
**Thalamus, ml; mean (STD)**
Baseline^a^	**15 (2.4)**	0.021	**14.03 (2.5)**	**16.5 (1.0)**	0.045	
3yr FU^a^	**14.8 (2.8)**		**13.97 (2.5)**	**16.2 (0.9)**	0.045	
**Caudate nucleus, ml; mean (STD)**
Baseline^a^	6.6 (1.0)		6.7 (1.2)	6.5 (0.7)		
3yr FU^a^	6.6 (1.1)		6.7 (1.3)	6.5 (0.6)		
**Putamen, ml; mean (STD)**
Baseline^a^	**8.33 (1.25)**	0.043	8.1 (1.4)	**8.7 (1.0)**		0.038
3yr FU^a^	**8.29 (1.26)**		8.0 (1.4)	**8.6 (1.0)**		
**Pallidum, ml; mean (STD)**
Baseline^a^	2.8 (0.3)		2.8 (0.4)	2.8 (0.3)		
3yr FU^a^	2.8 (0.3)		2.8 (0.3)	2.8 (0.3)		

*Volume data are given as mean (STD), all other parameters as median (range)*.

a *Values refer to the time point of the MRI scan at baseline and 3yr FU*.

*The p-values < 0.05 indicate significant results and are shown in brackets. Significant results are marked in boldface. BL, baseline; EDSS, Expanded Disability Status Scale; f, female; FLAIR, Fluid-attenuated inversion recovery; FU, follow-up; IRL, iron rim lesion; m, male; MP2RAGE, Magnetization Prepared RApid Gradient Echo; ml, milliliter; MSSS, Multiple Sclerosis Severity Score; r, range; RRMS, relapsing-remitting multiple sclerosis; SDMT: Symbol Digit Modality Test; SPMS, secondary progressive multiple sclerosis; yr, year*.

### Methods

#### MRI Acquisition and Processing

Imaging was performed according to our previous publication (7) on a Siemens Magnetom 7T MRI system, using a 32-channel radio frequency (RF) coil (Nova Medical, Wilmington, MA, USA). FLAIR images were acquired using a turbo spin echo (TSE) sequence with variable flip-angle echo trains. SWI data were acquired using a 3D fully first-order flow-compensated SWI sequence with TE = 25 ms, TR = 38 ms, image matrix = 704 × 704, slices = 96, resolution = 0.3 mm × 0.3 mm × 1.2 mm. Detailed information on pulse sequence parameters has been published before (50). Phase filtering and SWI image processing were performed by the manufacturer reconstruction. The FLAIR sequence was combined with the filtered SWI phase data (vendor-provided) as described previously, referred to as FLAIR-SWI contrast (55). T1-weighted data were acquired upon implementing an MP2RAGE sequence in 2018 with the following parameters: voxel size 0.375 mm × 0.375 mm × 0.75 mm, TI1/TI2 = 700/2,700 ms; TE = 4 ms, TR = 5,000 ms, flip angle1/2 = 4/5°, GRAPPA = 3, and a total acquisition of 8:03 min:s. Patients did not receive contrast-agent as we did not expect any current clinical benefit and due to reported concerns with contrast-agent applications (56, 57).

#### Lesion Identification and Quantification

Lesion selection criteria and quantification technique were consistent with our previous studies (7, 15). Supratentorial lesions of the periventricular, juxtacortical, and deep white matter in the frontal, parietal, and occipital lobes (58) and in the upper parts of the temporal lobes were analyzed. Due to field inhomogeneities near tissue–air interfaces, lesions in the inferior temporal lobes and infratentorially could not be adequately assessed. According to our publications (7, 15), segmentation was performed only for the lesions with well-demarcated borders. IRLs were defined as discrete hyperintense white matter lesions in FLAIR images, which were completely or partially surrounded by a hypointense rim seen on at least three contiguous slices in FLAIR-SWI. For the volumetry of non-IRLs, discrete non-confluent MS lesions (59) without dark phase rims but with distinct borders of FLAIR hyperintensity to surrounding normal-appearing white matter were selected. All lesions, including the 166 IRLs, were manually traced at baseline using display, part of the MINC toolbox (http://packages.bic.mni.mcgill.ca), by an MS expert with 10 years of experience in ultrahigh-field segmentation (ADB) under the supervision of a board-certified radiologist (ST). Assessment of intra-rater variability for lesion volumes was performed on 15 randomly selected IRLs and non-IRLs twice from the same rater (ADB) on FLAIR-SWI and MP2RAGE images. Intra-rater variability, based on the dice coefficient (DC) as a measure of the spatial overlap of two volumes, showed an “almost perfect match” (60). The DC ranges from 0 to 1, with 1 indicating perfect overlap. The DC (mean value ± SD) was calculated for FLAIR-SWI and MP2RAGE. DC values for FLAIR-SWI: pooled IRL and non-IRL = 0.89 ± 0.06, IRL = 0.92 ± 0.05, non-IRL = 0.86 ± 0.05, and for MP2RAGE: pooled IRL and non-IRL = 0.86 ± 0.14, IRL = 0.90 ± 0.02, and non-IRL = 0.83 ± 0.18.

#### Measurement of Lateral Ventricles and Subcortical Volumes

T1-weighted MP2RAGE images from two MRI scan points (baseline and 3yr FU) were analyzed using a robust automatic segmentation pipeline for 7T imaging as described below. The average time interval between the two MRI scans was 36.2 (±1.6) months. To increase data homogeneity, only patients who were scanned at both time points within 1 month were selected for this evaluation (*n* = 13). The demographic data of the atrophy cohort are summarized in Table 2. To prepare the T1w-MP2RAGE images for segmentation and to ensure consistent segmentation results across subjects, the six following pre-processing steps were performed:

1. Background noise removal using a regularized method of the combination of the two inversion time images (INV1 and INV2), resulting in corrected T1w-images without the strong background noise (61).
2. Lesion-filling with binary lesion maps using the Advanced Normalization Tools (ANTs, http://stnava.github.io/ANTs/).
3. Skull stripping using the HD-BET brain extraction tool (62).
4. N4 bias correction using a mask image in order to compensate for low-frequency image components only in the brain region (63).
5. Rician denoising of the T1w-images in order to reduce high-frequency Rician noise (64, 65).
6. Intensity normalization to the arbitrary range (0–100).

For subcortical segmentation, all images were resampled to 0.75 mm isotropic using tricubic interpolation. A single-subject template (SST) was then created using the ANTs template construction pipeline (66). This template represented the average morphology of the time series, which ensured an equally biased registration to each time point (65), which was also shown to increase segmentation consistency (67). Second, manual annotated labels based on 20 individual expert labels (68) were transformed into the SST image space (69). Third, the inverse subject-to-SST transformation for each time point was applied to the SST labels, warping the labels to each time point image. These labels were then used to determine the subcortical volumes using the ANTs LabelGeometryMeasures tool (66). Figure 1B shows an example of the segmentation results of the subcortical deep gray matter structures, in particular the lateral ventricles, thalamus, caudate nucleus, putamen, and pallidum, and was used for further analysis.

#### Analysis of Serum Neurofilament Light Chain Concentrations

For the measurement of sNfL concentrations, stored samples were thawed for 60 min at room temperature and analyzed by an investigator blinded to all clinical and MRI data using the Simoa Nf-light kits in the Simoa SR-X Analyzer (Quanterix, Lexington, MA, USA) (70). The sNfL assay was performed according to the instructions and protocol of the manufacturer and is described elsewhere (71). Briefly, thawed samples and calibrators were equilibrated to room temperature, diluted in sample diluent (1:4), and dispensed in 96-well plates as duplicates. About 20 μl of the detector and 25 μl of the paramagnetic beads were consecutively dispensed in each well, and plates were incubated and shaken (Simoa microplate incubator, 30°C, 800 RPM for 30 min). After pre-set washing steps on the Simoa microplate washer, 100 μl streptavidin β-galactosidase was added to each well, and plates were incubated (30°C, 800 RPM for 10 min) and washed. After a final washing step, plates were dried for 10 min before being transferred to the Quanterix SR-X analyzer for reading. Only samples with a coefficient of variance (mean sNfL concentrations as calculated by two replicates) of < 0.2 were included in this study. Statistical analysis of sNfL concentrations was based on tested blood samples from 27 patients for each time point.

#### Neuropsychological Examination

**Figure 3:**
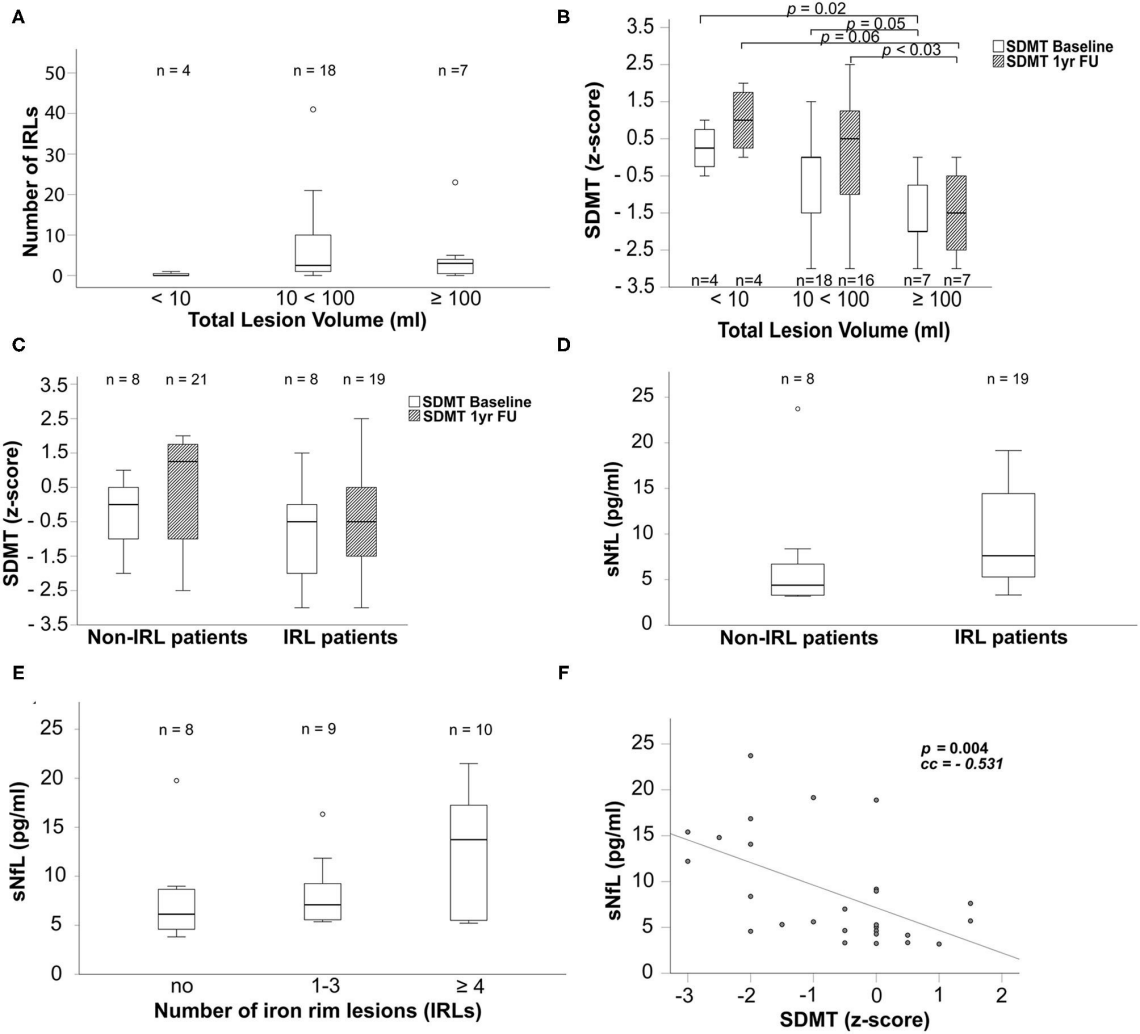
Boxplots display in **(A)** the number of iron rims in patients grouped by their MP2Rage total lesion volume in ml, in **(B)** SDMT *z*-scores grouped according to the MP2Rage total lesion volume in ml at baseline (white bars) and 1yr FU (cross-hatched bars), in **(C)** SDMT *z*-scores grouped according to non-IRL and IRL patients at baseline (white bars) and 1yr FU (cross-hatched bars), in **(D)** sNfL concentrations in pg/ml in non-IRL and IRL patients and in **(E)** sNfL concentrations in pg/ml grouped according to count of IRLs. **(F)** The scatter plot reflects the relationship between sNfL concentrations and the SDMT. Number of evaluated patients is given within the graphs. The *p-*values of boxplots indicate results from non-parametric Kruskal–Wallis tests and *post-hoc* Mann–Whitney *U-*tests. The *p-*value of the scatterplot indicates the Spearman's rank correlation coefficient used to evaluate the bivariate correlation. Only significant *p-*values are given. IRLs, iron rim lesions; FU: follow-up; SDMT, symbol digit modalities test; sNfL, serum neurofilament light chain; yr, year.

The SDMT was assessed by clinical neuropsychologists in 29 MS patients at baseline and in 27 of those patients 1 year thereafter. Two patients did not participate in the neuropsychological follow-up. The neuropsychological performance is presented as SDMT *z*-score in mean and SE according to the IRL presence (Table 3; Figure 3C). The change of neuropsychological performance in SDMT within 1 year was compared between IRL and non-IRL patients and given as the *p*-value in Table 3. Furthermore, SDMT z-scores were presented according to the lesion volume (Figure 3B) and sNfL (Figure 3B).

**Table 3:** Change of SDMT *z*-scores within 1 year between IRL and non-IRL patients of the total cohort.

	**Change within 1 year IRL vs non-IRL**	**IRL patients (BL: *n* = 21;1yr FU: *n* = 19)**	**Patients with non-IRL (BL: *n* = 8; 1yr FU: *n* = 8)**
		**BL/1yr FU**	**BL/1yr FU**
	***p*-values**	**mean ± SE**	**mean ± SE**
**SDMT** ***z*****-score**	0.243	−0.76 ± 0.28/−0.47 ± 0.34	−0.25 ± 0.40/0.44 ± 0.66

*Values are given in mean (SE)*.

*The p-value < 0.05 indicates significant result*.

*BL, baseline; FU, follow-up; SDMT: Symbol Digit Modalities Test*.

#### Statistical Analysis

Data were analyzed using the IBM SPSS®Statistics for Windows Version 26 (IBM Corp., Armonk, NY, USA). Paired *t*-tests (for normally distributed differences) or Wilcoxon matched-pairs signed ranks tests (for skewed differences) were used to test for changes over time. Mann–Whitney *U*-tests (to compare two groups) or Kruskal–Wallis tests (for three or more groups) were used to compare groups with respect to volumes, sNfL concentrations, and neuropsychological test results. Boxplots were used for graphical representation. Because of skewed data, Spearman's rank correlation coefficient was used to evaluate bivariate correlations. The *p*-values ≤ 0.05 were considered significant.

## Results

Demographic, clinical, and MRI data are given in Tables 1, 2. At the beginning of the MRI examination, one patient developed a brief episode of claustrophobia, and 2 patients reported mild dizziness when entering the scanner. No MRI scan had to be interrupted or aborted for these reasons. No intravenous contrast agent was administered.

### Patients With IRLs Show More FLAIR Lesions

Based on MRI scans at baseline, 1,013 hyperintense supratentorial FLAIR lesions were detected in our MS cohort, of which 16.4% (*n* = 166) showed a surrounding SWI-detected hypointense iron rim (IRL). About 27.6% of patients (*n* = 8/29) had no IRL, 37.9% of patients (*n* = 11/29) had 1–3 IRLs, and 34.5% of patients (*n* = 10/29) had more than four IRLs. As shown in Table 1, IRL patients (*n* = 21) had significantly more FLAIR lesions than non-IRL patients, regardless of the number of IRLs according to the above-mentioned groups [*p* = 0.047; IRL patients: 32 (5–98) vs. non-IRL patients: 11.5 (2–64)]. MP2RAGE lesion count [*p* = 0.09; IRL patients: 23 (3–115) vs. non-IRL patients: 11.5 (1–135)] and MP2RAGE lesion volume in ml [*p* = 0.2; IRL patients: 31.4 (2.4–241.9) vs. non-IRL patients: 14.4 (0.8–439.9)] did not differ significantly between IRL and non-IRL patients.

Patients with an MP2RAGE lesion volume between 10 and 100 ml (*n* = 18) had significantly more IRLs than patients with less than 10 ml (*n* = 4) (*p* <0.014; Figure 3A). Due to the comparison of 4 patients with 18 patients, we omitted the *p*-value in Figure 3A. As shown in Figure 3B, higher MP2RAGE lesion volume was associated with lower cognitive performance (SDMT) at baseline and after 1 year. Patients with an MP2RAGE lesion volume higher than 100 ml showed significantly worse SDMT performance compared to the patients with lesion volume of 10–100 ml (BL*: p* = 0.05, 1yr FU: *p* = 0.03) and below 10 ml (BL: *p* = 0.02; 1yr FU: *p* = 0.06) [SDMT baseline: <10 ml: 0.25 (−0.5 to 1); 10 <100 ml: 0 (−3 to 1.5); >100 ml: −2 (−3 to 0); SDMT 1yr FU: <10 ml: 1 (0–2); 10 <100 ml: 0.5 (−3 to 2.5); >100 ml: −1.5 (−3 to 0)]. IRL patients did not show significantly worse SDMT performance than non-IRL patients (Figure 3C; BL: *p* = 0.218; 1yr FU: *p* = 0.106, even when grouped by the number of IRLs (0; 1–3; ≥4 IRLs) (BL: *p* = 0.236; 1yr FU: *p* = 0.253).

### Patients With IRLs Show Thalamic Atrophy

The results presented are based on the described segmentation pipeline on 7T image data used to measure the subcortical structures (thalamus, caudate nucleus, putamen, and pallidum) and lateral ventricle volumes on 7T T1w-MP2RAGE images at baseline and 3 years later. Detailed demographic, clinical, and MRI data are shown in Table 2. Within 3 years, the atrophy cohort showed a significant shrinkage of thalamic volume (Figure 1C; *p* = 0.021; BL: 15 ± 2.4 ml; 3yr FU: 14.8 ± 2.8 ml) and the putamen volume (*p* = 0.043; BL: 8.33 ± 1.25 ml; 3yr FU: 8.29 ± 1.26 ml), and significant enlargement of the lateral ventricles (Figure 1D; *p* = 0.012; BL: 20.6 ± 7.1 ml; 3yr FU: 22.2 ± 8.4 ml). Such changes were also observed in non-IRL patients for the lateral ventricles (*p* = 0.045; BL: 17.3 ± 3.5; 3yr FU: 19.1 ± 4.8) and the putamen (*p* = 0.038; BL: 8.7 ± 1.0; 3yr FU: 8.6 ± 1.0; Table 2). Interestingly, the thalamic volume of IRL patients was significantly lower compared to non-IRL patients at both time points (BL: *p* = 0.045; 3yr FU: *p* = 0.045). Loss of thalamus volume within 3 years was significantly higher in IRL than in non-IRL [*p* = 0.019; IRL: −0.29 ml (−0.79 to 0.02) vs. non-IRL: −0.06 ml (−0.19 to 0.10)].

### Serum NfL Concentrations Are Higher in Patients With IRLs

Blood samples were obtained at baseline and after 3 years. Table 1 shows the median concentration levels and ranges of sNfL in the total cohort and grouped by the presence of IRL for both time points, baseline and 3yr FU. The sNfL concentration increased in all groups over 3 years [total cohort: BL: 5.7 pg/ml (3.2–23.7), 3yr FU: 8.0 pg/ml (3.8–21.5); IRL patients: BL: 7.6 pg/ml (3.3–19.2), 3yr FU: 8.8 pg/ml (5.2–21.5); non-IRL patients: BL: 4.4 pg/ml (3.2–23.72); 3yr FU: 6.1 pg/ml (3.8–19.8)]. IRL patients had higher concentrations of sNfL at both time points, with significantly higher concentrations at baseline compared to non-IRL patients (Figure 3D, *p* = 0.045). The *p*-value was omitted from Figure 3D because of the small number of patients. Concentrations of sNfL increased with the number of IRLs without significant differences between groups (Figure 3E). Within 3 years, sNfL concentrations also did not increase significantly in any of the cohorts, as shown in Table 1 (total cohort: *p* = 0.225; IRL patients: *p* = 0.051; non-IRL patients: *p* = 0.23). For relative changes, we defined an increase in sNfL concentration of more than 25% and a decrease of 20% from baseline as the ratio of 100:125 equals the ratio of 80:100. In the total cohort, sNfL remained stable in 53.8% (14/26), increased in 38.5% (10/26), and decreased in 7.7% (2/26). IRL-patients showed stable sNfL levels in 50% (9/18), increased in 38.9% (7/18), and decreased in 11.1% (2/18). The sNfL of non-IRL patients remained stable at 62.5% (5/8) and increased in 37.5% (3/8).

### Serum NfL Concentrations Correlate With Neuropsychological Impairment in IRL and Non-IRL Patients

As shown in Figure 3F, high sNfL concentrations were associated with lower SDMT performance in the total cohort (*p* <0.004), IRL patients (*p* <0.034), and non-IRL patients *(p* <0.030).

### Patients With IRLs and Non-IRLs Do Not Differ Significantly in Change in Neuropsychological Performance After 1 Year

Patients with IRLs performed slightly worse on the SDMT at baseline and 1 year, yet there was no significant difference between mean *z*-scores or between changes in neuropsychological performance within 1 year compared with non-IRL patients (Table 3 and Figure 3C).

## Discussion

Our exploratory pilot study focuses on the clinical, radiological, and serological differences between patients with and without IRLs to understand the further significance of IRLs, a promising imaging biomarker for severe disease course and disease progression in MS (8, 9). We tested our 4 primary defined objectives: lesion load, volumes of subcortical structures and lateral ventricles, sNfL, and the SDMT, as we hypothesized a worse outcome of IRL patients compared to non-IRL patients. The following 3 findings support the destructive potential of IRLs: First, IRL patients have a significantly higher number of FLAIR lesions. Second, IRL patients showed significantly smaller thalamic volume. Third, IRL patients showed significantly higher sNfL concentrations, at least at the baseline.

There was no significant difference in performance on the SDMT within 1 year between IRL and non-IRL patients. Nevertheless, all results discussed here are preliminary findings from our small pilot study, but are essentially consistent with the destructiveness of iron rims reported in the literature.

### Patients With IRLs Show More FLAIR Lesions

This finding should lead to a closer examination of IRLs as biomarkers of high lesion burden, indicating a more severe disease course and providing a further argument for early therapy, as demonstrated in a long-term study in which MS patients with an initial high FLAIR lesion burden had greater clinical disability after 20 years than MS patients with a low initial FLAIR lesion burden (6). However, it has been previously shown that not only the number but also the extent of T1-hypointensity within FLAIR lesions on the fast FLAIR image at 1.5T is significantly more pronounced in patients with SPMS and correlates with EDSS (72). This was prior to technical advances in high-field MRI and iron rim imaging with susceptibility-sensitive MRI sequences such as SWI, R2^*^, or QSM and may have already demonstrated the demyelinating potential and/or lack of remyelination of IRLs, which are reflected as T1-black holes and contribute to severe tissue loss (17, 73). IRLs might therefore play a potential role in the future as a vanguard predictor of high lesion burden associated with clinical deterioration (9), leading to disability at an earlier age (8).

### Patients With IRLs Show Thalamic Atrophy

Brain atrophy results from years of chronic inflammation and axonal degeneration on top of physiological brain volume loss with age. However, it is also already found early in MS (74–77) but is most pronounced with disease duration (78, 79). It is reported to correlate more closely with the clinical impairment than the T1 or T2 lesion load in the white matter and identifies patients at risk for progression (80), making it an important MRI biomarker (81, 82). In particular, the gray matter volume loss is of increasing interest as it is more strongly associated with the clinical impairment than the white matter atrophy (83–87). Among gray matter structures, the thalamus is considered particularly susceptible to neurodegeneration through lesions within the thalamus itself, but also indirectly *via* the crossing demyelinated nerve tracts of the white matter and cortex, and therefore serves as a measure of diffuse parenchymal damage (88). Thalamic volume changes have been reported to be associated with cognitive impairment (89–91). This would be consistent with our results showing significant thalamic volume loss within 3 years in the atrophy cohort. Here, we would like to emphasize that the patients with IRLs in this cohort (*n* = 8) had a significantly smaller thalamus, which is consistent with a cross-sectional study of 192 patients that showed significant volume loss in the basal ganglia (thalamus, caudate nucleus, and putamen), especially in patients with more than 4 iron rims (8). In contrast to Absinta et al. (8), the significant increase in lateral ventricles was only detected independently of IRLs, possibly due to the small number of patients. However, our results reflect atrophy within 3 years of observation, with particular focus on the significantly small thalamus in IRL patients, whose dynamics will be monitored in the next years.

### Serum NfL Concentrations Are Higher in IRL Patients

Disanto et al. (36) found higher sNfL levels in patients with active Gd-enhancing MS lesions in both the brain and spinal cord, whose pathology particularly contributes to clinical deterioration. The fact that, in addition to age, relapses and disability as measured by EDSS were positively and independently associated with higher sNfL levels already suggested that higher sNfL levels reflect not only active inflammation but also chronic, slowly progressive inflammation that eventually leads to disability progression. Now, a recent study (44) has shown that high sNfL levels are significantly driven by chronic, active IRLs, independent of all known factors affecting sNfL (e.g., T2 lesion load, disease course, EDSS, and DMTs). Higher atrophy rates and earlier clinical deterioration in IRL patients (8) are therefore now further supported by prominent neuroaxonal damage reflected in higher sNfL levels than in non-IRL patients (44). This strong association between IRL and sNfL suggests IRL—an imaging marker of chronic active inflammation—as a significant driver of neuroaxonal damage without evidence of acute inflammation (44). In line with this recent study (44), we also found that concentrations of sNfL increased with the number of IRLs. Furthermore, higher sNfL concentrations were associated with poorer neuropsychological performance in our study population, irrespective of IRLs. The proportions with a significant sNfL increase (>25%) in our study were higher in the IRL cohort than in the non-IRL cohort. This increase even exceeded the age-matched 1% per year increase as shown in a large cohort by Khalil et al. (41). Our data are specifically interesting as IRL and non-IRL patients in our study cohort were of comparable median age and disease duration, which rule out the influence of age and disease duration on sNfL concentration in our small cohort [age: IRL: 38 years (22–60) vs. non-IRL: 36 years (22–69); disease duration: IRL: 11 years (5–37) vs. non-IRL: 12.5 years (6–40)]. In addition, the individual age of the patients was comparably distributed within the groups. Furthermore, the group of non-IRL patients even included the oldest study patient and had a slightly longer disease duration. As the distinction between normal and pathological aging based on sNfL levels is not yet reliable (41) and a normative database is lacking (49), internal control within our demographic comparable group appears favorable and reliable. Since the patients in our study did not receive contrast agent, no correlation would have been possible here. Patients who received different therapies, such as rituximab, natalizumab, fingolimod, siponimod, and ocrelizumab in randomized trials, were reported to have significantly lower sNfL levels compared to the placebo group (49). Due to the small study cohort and differences in patient therapy, no analysis could be performed here. Nevertheless, according to Maggi et al. (44), IRLs were found to be a significant and apparently independent driver of neurodegeneration, as reflected by high sNfL levels. This has shed light on sNfL in chronic inflammation from a new perspective without the significant importance of the known factors influencing sNfL.

The increased axonal damage assessed by sNfL in IRL patients complements the knowledge of the destructiveness of IRLs (17) in terms of their association with higher lesion burden, atrophy, prominent black holes, and the worse clinical and neuropsychological outcome described earlier (8, 9). Our findings, therefore, are consistent with chronic inflammation and increased sNfL (44) and with the reported associations between increased sNfL, increased lesion burden, and atrophy (14, 42). Furthermore, neurofilament is assigned a value as a predictor of long-term clinical outcome (12) and is thought to be weakly associated with neuropsychological impairment (48). All these NfL-associated parameters fit the destructive lesion characteristics of IRLs described above.

### Patients With IRLs and Non-IRLs Do Not Differ in Neuropsychological Performance Within 1 Year

Patients with MS often suffer from neuropsychological deficits. They can be easily missed, particularly in the very early stages in patients with stable EDSS, and become more and more pronounced in the progressive stage according to the proceeding atrophy (92, 93). Explicit neuropsychological deterioration in patients with RRMS is discussed as a conversion marker into the progressive stage of MS as a result of structural damage over time with increasing cognitive dysfunction causing the network to collapse (94). Therefore, neuropsychological testing at the onset of the disease is considered to be of great importance. We tested the total cohort at 1-year intervals on SDMT, particularly a sensitive test to detect slow information processing commonly seen in MS (54). IRL and non-IRL patients showed no significant differences in their neuropsychological performance within 1 year. Therefore, we extend our neuropsychological follow-up tests to the third and fifth years.

Since our study results point substantively in the same direction as other IRL studies (8, 44), we see preliminary value in our findings, but they definitely need to be confirmed in multiple larger cohort studies.

## Limitations

This study has some limitations. First, our 7T MRI long-term exploratory pilot study includes only a relatively small number of patients. Therefore, we did not take potential covariates related to sNfL (e.g., age, disease duration, MRI lesion load, and treatment) into account to avoid further subgroup reduction. However, on the one side, we consider the group comparison to be a reliable approach because of the comparability in age and disease duration of the IRL and non-IRL patients; and on the other side, according to Maggi et al. (44), the association between sNfL and IRL was significant regardless of known factors influencing sNfL. Second, the interval between MRI and blood sampling only at baseline averaged 7.5 ± 2.7 months. At the 3yr FU time point, MRI and blood sampling were performed on the same day. Nevertheless, given the evidence that PRLs have been shown to last at least 7 years (15), IRL and non-IRL groups are considered as stable. Furthermore, sNfL values of our cohort remained also quite stable over time. Moreover, the significant result of sNfL levels between the IRL group and the non-IRL group is based on blood sampling at the same time point. Serum and MR parameters in our study were independently pointing toward pronounced neurodegeneration in the IRL group as previously shown (8, 44). We further consider the only 1-year observation period of neuropsychological performance too short, which should have initially served as a clinical differentiator in our relatively EDSS stable MS cohort. Nevertheless, our tested objectives were already in this small cohort significantly different between IRL and non-IRL patients, implying from a statistical point of view that the differences must be large. Moreover, the significant results fit very well the literature on the destructive nature of IRLs and go in line with a larger cohort study (44). We continue to monitor our cohort for lesion number, atrophy, neurofilament dynamics, and neuropsychological deficits associated with the presence of IRLs.

## Conclusion

With our pilot study, we aim to contribute to a more accurate knowledge of IRLs in MS. Our current results confirm that patients with IRLs have more FLAIR lesions, show signs of thalamic atrophy, and have higher concentrations of sNfL compared with non-IRL patients. Therefore, our preliminary findings point in the direction of the discussed destructive nature of IRLs. In the future, the combined power of serum and imaging biomarkers, in addition to clinical parameters, may facilitate the assessment of the clinical risk profile and thus play a role in the future therapy management. Additional long-term observations of atrophy, sNfL concentrations, and neuropsychological performance in clinical trials with a representative number of patients with and without IRLs would be an important step to further assess the neurodegenerative properties of IRLs.

## Data Availability Statement

The authors are willing to provide data on this manuscript upon reasonable request.

## Ethics Statement

The studies involving human participants were reviewed and approved by the Ethics Committee of the Medical University of Vienna (EC 154/2009). The patients/participants provided their written informed consent to participate in this study.

## Author Contributions

All authors contributed to the study design, data acquisition, data analysis, data interpretation, manuscript drafting, and approved the final version.

## Funding

This project was supported by our Medical University as well as by an unrestricted grant from Merck Gesellschaft mbH, an affiliate of Merck KGaA, Darmstadt, Germany (MS200136_0051). The funder of the study had no influence on study design, data collection, data analysis, data interpretation, or writing the manuscript or in the decision to submit for publication. None of the authors were paid by the company to write this article.

## Conflict of Interest

The authors declare that the research was conducted in the absence of any commercial or financial relationships that could be construed as a potential conflict of interest.

## Publisher's Note

All claims expressed in this article are solely those of the authors and do not necessarily represent those of their affiliated organizations, or those of the publisher, the editors and the reviewers. Any product that may be evaluated in this article, or claim that may be made by its manufacturer, is not guaranteed or endorsed by the publisher.

## Abbreviations

DC: dice coefficient
DMT: disease-modifying therapy
EDSS: Expanded Disability Status Scale
FLAIR: Fluid-attenuated inversion recovery
GD^+^T1: gadolinium positive signal on T1
GRAPPA: GeneRalized Autocalibrating Partial Parallel Acquisition
IRL: iron rim lesion
MP2RAGE: magnetization prepared rapid gradient echo
MS: multiple sclerosis
MSSS: multiple sclerosis severity score
9-HPT: nine-hole peg test
QSM: quantitative susceptibility mapping
R2^*^: inverse of the transverse relaxation rate T2^*^ (1/T2^*^)
RF: radio frequency
RRMS: relapsing-remitting multiple sclerosis
RPM: rounds per minute
SDMT: symbol digit modalities test
sNfL: serum neurofilament light chain
SPMS: secondary-progressive multiple sclerosis
SST: single-subject template
SWI: susceptibility weighted imaging
T: Tesla
T1w: T1-weighted
TE: time to echo
T25FW: timed 25-foot walk test
TI: inversion time
TR: repetition time
TSE: turbo spin echo.
